# Elevated Linoleic Acid (A Pro-Inflammatory PUFA) and Liver Injury in a Treatment Naive HIV-HCV Co-Infected Alcohol Dependent Patient

**DOI:** 10.4236/jbm.2016.47003

**Published:** 2016-07

**Authors:** Vatsalya Vatsalya, Shirish S. Barve, Craig J. McClain, Vijay A. Ramchandani

**Affiliations:** 1 Division of Gastroenterology, Hepatology and Nutrition, Department of Medicine, University of Louisville School of Medicine, Louisville, USA; 2 Section on Human Psychopharmacology, LCTS/DICBR/NIAAA at National Institutes of Health, Bethesda, USA; 3 Department of Pharmacology and Toxicology, University of Louisville School of Medicine, Louisville, USA; 4 Robley Rex VA Medical Center, Louisville, USA

**Keywords:** Alcohol, Fatty Acids, HCV, HIV, Liver Injury

## Abstract

HIV and HCV co-infection is a unique disease condition, and medical management of such condition is difficult due to severity and systemic complications. Added with heavy alcohol drinking, risk of liver injury increases due to several pro-inflammatory responses that subsequently get involved with alcohol metabolism. Elevated levels of fatty acids have been reported both in viral infections as well as alcoholic liver disease though such investigations have not addressed the adverse events with dual viral infection of HIV and HCV along with heavy drinking. This case report is of a patient with excessive alcohol drinking and first time diagnosis of HIV and HCV dual infection, elaborating concurrent alteration in Linoleic Acid (LA) levels and pro-inflammatory shift in *ω*-6/*ω*-3 ratio along with the elevations in liver injury markers. Elevated LA has been recently studied extensively for its role in alcoholic liver disease; and in the present case, we also found it to be clinically relevant to liver injury.

## 1. Introduction

HCV infection is found in almost 50% of the HIV-infected individuals in the US (largely due to intravenous drug abuse), which may lead to fast progression to end-stage liver disease [1]. Viral infections have shown higher morbidity and mortality in patients who also drink heavily due to exacerbated immune dysregulation and clinical developments in liver both as a result of several pathological processes targeting liver condition as well as due to the adverse effects of antiretroviral (ART) therapy [2]. Therefore, it is essential that providers, working with such special patient population who also drink heavily, be able to fully characterize the onset of liver injury supported by the knowledge of risk factors during the course of the disease for appropriating medical management. Several risk factors have been identified previously in this co-infected population, for example, HCV viral load, Ferritin levels, Body Mass Index [3] though more studies are needed to determine the markers with sensitivity to characterize early indication of liver injury when such patients report about heavy drinking history. Arrangements of fatty acids (FAs) participating in inflammation during liver injury due to heavy drinking [4] has been found as viable indicator of liver injury, and changes in the arrangement of the FAs could be an useful marker of liver injury in such complex comorbidity. A case study on treatment naïve HIV diagnosis in an alcohol dependent patient who was also confirmed with HCV infection is presented to discuss the changes in fatty acid panel and liver injury.

## 2. Case Presentation

The patient was a 41-year-old widowed male at the time of screening who started drinking at age eight and progressed to become a regular drinker by age 16. At screening, he reported heavy drinking for the last ten years and had a positive family history of alcoholism. He reported excessive drinking (one-two pints of hard liquor daily, [8.5 - 17 drinks]) after a major life event occurred as the death of his spouse (approximately eight months prior to admission). His BMI was 22; he reported heavy smoking (one pack daily), and recent involvement in unsafe lifestyle practice. He showed confirmatory signs of alcohol use disorder with withdrawal symptoms during the psychiatric evaluation. Patient also reported that he was never tested positive for viral infection or taken medication for its treatment during intake. This patient was diagnosed with positive HCV and HIV viral tests during the screening. HCV RNA IU quantification was at 8,920,000 with genotype 1A. HIV infection was detected based on both the criteria for confirmation for HIV infection as developed by the Center for Disease Control; with HIV-1 Ab detection, and western blot assay at viral load of >50. His sCD4 count was 210, which put him in the category 2 according to the CD4+ T-Lymphocytic classification. The test was reconfirmed with western blot band with second positive test at bands p24, gp41, and gp120/160. There was no other remarkable systemic observation and he was not diagnosed with any other relevant medical condition, other than the discussed drug abuse and viral infection. This patient presented asymptomatic conditions of HIV infection (resembling criteria for “clinical category A”).

**Figure 1:**
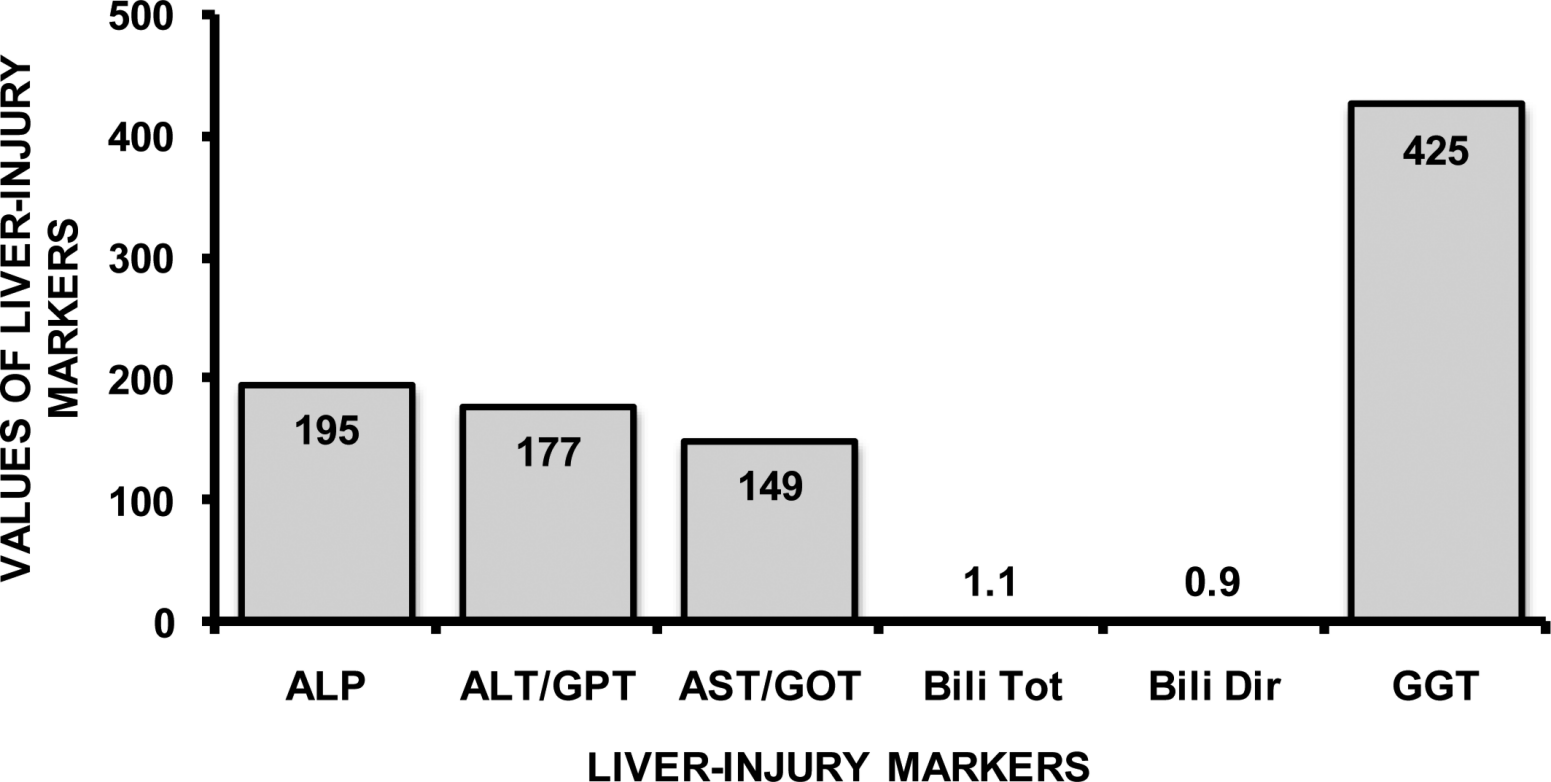
Elevation in Liver-injury in heavy drinking patient diagnosed with HIV and HCV co-infection. ALP: Alkaline Phosphatase (normal range: 37 - 116 U/L); ALT/GPT: Alanine Transaminase (normal range: 6 - 41 U/L); AST/GOT: Aspartate Transaminase (normal range: 9 - 34 U/L); Bili Tot: Bilirubin, Total (normal range: 0.1 - 1.0 mg/dL); Bili Dir: Bilirubin Direct (normal range: 0.0 - 0.2 mg/dL); GGT: Gamma Glutamyltransaminase (normal range: 5 - 85 U/L).

This patient presented with a remarkable liver injury profile (**Figure 1**), with all the liver injury markers showing above the normal values. In co-infected HIV+HCV cases, even with severe liver fibrosis and cirrhosis, 10% to 25% patients have shown normal alanine aminotransferase (ALT) levels [5]. Therefore, this case seems to show added severity in liver injury due to heavy alcohol drinking. Also, clinically relevant complete blood count (with differential) was identified at low hemoglobin (13.3, normal: 13.7 - 17.5 g/dL), hematocrit (37.2, normal: 40.1% - 51.0%) and MCV (78.6, normal: 79.0 - 92.2 fL) levels showing suppression of blood cell production this state of the infection. Ferritin was identified as high, 408 (26 - 388) as is generally observed in infections. Abnormal levels of leucocytes (WBC: 146/mcl; normal 0 - 25) were present and specific subtypes namely monocytes and lymphocytes [6] as shown in **Table 1**. In this patient, fatty acid panel tested with blood (plasma) specimen showed that linoleic acid was the only PUFA that was abnormally high at 3957 nmol/mL (normal range: 2270 - 3850) that has shown important role in liver injury [7]. Both HCV and HIV cause dysregulation of lipid metabolism, especially in polyunsaturated fatty acids (PUFAs) [8]. We also evaluated the *ω*-6/ *ω*-3 ratio, which was 18 (Total *ω*-3 was 0.3; and Total *ω*-6 was 5.4, units mmol/L) in this patient supporting significant increase in the pro-inflammatory shift [9]. Triene Tetraene ratio was at the lower margin at 0.01 (normal range: 0.010 - 0.038). No other clinically significant change was observed in the FAs involved in inflammation; or in the lipid panel. Immunoglobulin panel at this point was not abnormal. Patient received adequate medical management for alcohol withdrawal during the screening process [10] and was referred for infectious disease specialty.

## 3. Discussion

LA was significantly high in this patient and this case showed the relevance of analyzing the fatty acid panel with respect to liver injury. A pro-inflammatory response might be a useful measure to evaluate liver injury in patients who drink heavily and are diagnosed with viral co-infection. Early detection of viral infection could result in better medical management of the disease however few studies have evaluated alcohol drinking and their involvement in inflammation in such specific patient population. Involvement of elevated LA and subsequent changes in the fatty acids have been reported along with the pro-inflammatory and anti-inflammatory responses [11]. Understanding of pro-inflammatory response with the changes in *ω*-6 FAs could further elucidate the state and potential progression of liver injury in such cases. A similar study reported that lifetime drinking and Hepatitis C virus infection play role in the progression of liver disease [12], however it did not discuss about the fatty acids and their involvement in inflammation and liver injury. More studies are needed on specific fatty acids [13] for their role in inflammation; and their use as dietary supplements and metabolic responses are needed to investigate regulation and reduction of inflammation causing liver injury in this unique morbidity. This becomes much more relevant since ART therapy for HIV infection is known to cause liver injury, and our case report adds value to effective management of liver disease and treatment outcomes in HIV and HCV co-infected AD patients by having pre-treatment evaluation of pro-inflammatory response that might be adversely influencing the liver condition.

## 4. Conclusion

Liver injury is a known harmful outcome of heavy drinking and manifestation in HCV infection. Clinically relevant level of linoleic acid seems to correspond with the severity in liver injury in this dual viral infection that is likely getting negatively impacted by heavy alcohol intake as well, supporting involvement of ongoing pro-inflammatory response. Additional testing for comprehensive fatty acid panel could elucidate the ongoing changes in inflammatory response. In our patient, this was primarily influenced by increase in the ω-6 fatty acid, and was noted by the elevation in linoleic acid level. Patients with HIV and HCV co-infection and excessive alcohol intake might need additional evaluation for liver state, before the start of ART treatment. Linoleic acid might serve as a marker of pro-inflammatory response along with the ω-6/ω-3 ratio that likely could explain the severity in liver injury. Medical management of such cases becomes more challenging due to a pro-inflammatory response adversely effecting liver and limiting choices of antiretroviral therapy specific to HIV that also causes liver injury.

## Figures and Tables

**Table 1:** Measures from differential CBC for White Blood Cell shows some significant elevation in macrophages (Lymphocytes and Monocytes) and Neutrophils (Polys) showing neutropenia. Normal ranges and measure units are based on the guidelines provided by National Institutes of Health and Mayo Clinic.

CBC Differential	Values	Normal Ranges	Units
Lymphocytes	**56.8 ↑**	**21.8 - 53.1**	**%**
Monocytes	**14.6 ↑**	**5.3 - 12.2**	**%**
Eosinophils	**4.6**	**0.8 - 7.0**	**%**
Basophils	**0.9**	**0.2 - 1.2**	**%**
Polys Absolute	**1.06 ↓**	**1.78 - 5.38**	**K/uL**
Lymphocytes Absolute	**2.6**	**1.32 - 3.57**	**K/uL**
Monocytes Absolute	**0.67**	**0.30 - 0.82**	**K/uL**
Eosinophils Absolute	**0.21**	**0.04 - 0.54**	**K/uL**
Basophils Absolute	**0.04**	**0.01 - 0.08**	**K/uL**

## Abbreviations

AD: Alcohol Dependents
ALT: Alanine aminotransferases
CS: Clinically significant
FA: Fatty Acid/s
HIV: Human Immunodeficiency Virus
LA: Linoleic Acid
NCS: Clinically non-significant
PUFAs: Polyunsaturated fatty acids
LDH: Lifetime drinking history
